# Short-Duration Beta-Alanine Supplementation Did Not Prevent the Detrimental Effects of an Intense Preparatory Period on Exercise Capacity in Top-Level Female Footballers

**DOI:** 10.3389/fnut.2020.00043

**Published:** 2020-04-21

**Authors:** Rafael Ribeiro, Breno Duarte, André Guedes da Silva, Guilherme Passos Ramos, Andreia Rossi Picanço, Eduardo Macedo Penna, Victor Coswig, Matheus Barbalho, Paulo Gentil, Bruno Gualano, Bryan Saunders

**Affiliations:** ^1^Applied Physiology and Nutrition Research Group, School of Physical Education and Sport, Rheumatology Division, Faculdade de Medicina FMUSP, Universidade de São Paulo, São Paulo, Brazil; ^2^Confederação Brasileira de Futebol, Rio de Janeiro, Brazil; ^3^Physical Education, Federal University of Pará – Campus Castanhal, Castanhal, Brazil; ^4^Physical Education and Dance Institute, Federal University of Goiás, Goiânia, Brazil; ^5^Institute of Orthopaedics and Traumatology, Faculty of Medicine FMUSP, University of São Paulo, São Paulo, Brazil

**Keywords:** football training, nutritional supplementation, YoYo intermittent recovery test, repeated sprints, competition, fatigue, elite

## Abstract

**Purpose:** High-intensity activity is an important aspect of football performance during competitive match play. The aim of this study was to investigate the effect of beta-alanine supplementation throughout a short-duration intense football-specific training period prior to an international competition on measures of high-intensity running performance.

**Methods:** Twenty-four elite international U20 female footballers (age 18 ± 1 y, height 1.67 ± 0.07 m, body mass 62.7 ± 7.4 kg) volunteered to perform the YoYo Intermittent Recovery Test Level 1 (YoYo IR1), the Running Anaerobic Sprint Test (RAST) and a 20-m maximal sprint test on two separate occasions, separated by 3 weeks of training and supplementation. Participants were randomly assigned to receive either 6.4 g·day^−1^ sustained-release beta-alanine (BA, *N* = 12) or an equivalent dose of maltodextrin (placebo, PL, *N* = 12) throughout a 3-week standardized training camp.

**Results:** There was a main effect of group (*P* = 0.05) and time (*P* = 0.004) on YoYo IR1; overall values were lower in PL and distance covered was lower post- vs. pre-supplementation. There was no group × time interaction (*P* = 0.07). There was an effect of sprint number for RAST, but no further main effects and there were no effect for the 20-m sprint.

**Conclusions:** Top-level female footballers involved in this intense 3-week training period prior to a competition worsened their high-intensity intermittent exercise capacity, and this negative result was not attenuated by a short-duration BA supplementation protocol throughout the same period. Further work is necessary to elucidate whether adapted training protocols and BA dosing regimens could lead to better results.

## Introduction

Football, also commonly termed as soccer, is the world's most popular sport, practiced by men and women all around the world (1). The women's game has seen a stark increase in both popularity and professionalism over the past decade, with research into the physiological demands of the women's game following a similar rise in popularity (2). The general characteristics of women's football demonstrate that match-play is predominantly performed at low-intensity activities interspersed by numerous high-intensity actions throughout (2, 3). Key moments that can affect the outcome of a game generally occur at high-intensities. High-intensity efforts are reduced during various phases of international matches and vary according to position (4) and top-level women players have been shown to perform more high-intensity running and sprints during games than their less successful counterparts (5). High-intensity activity, therefore, appears to be an important aspect of football performance during competitive match play.

Several field tests are employed to evaluate the training status of football players and are commonly used to predict match performance and determine the effectiveness of a training intervention. The YoYo Intermittent Recovery Tests (Level 1 [YoYo IR1] and 2 [YoYo IR2]) evaluate an individual's capacity to repeatedly perform and recover from intense exercise bouts, and is applicable to team sports players due to the specificity of the exercise undertaken (6). The Yo-Yo IR1 and IR2 have been shown to correlate to various variables of match performance and can be used as an indicator of the physical performance of elite female players throughout competitive matches (7, 8), making them appropriate models to examine the effect of any intervention designed to manipulate changes in performance during team sports. In addition to differentiating between playing standard, these tests can be used to monitor training adaptations, seasonal variation and determine differences between playing position [for review, see (9)]. The running anaerobic sprint test (RAST) is another protocol that has been shown to be reliable and valid to assess anaerobic power and is a good predictor of short-distance running performances (10) while the 20-m sprint test is a commonly used measure to assess team sport players (11). Any changes in these performance measures may be reflective of an enhanced capacity to improve in-match performance and thus are useful tools to determine the efficacy of any intervention.

The preparation period prior to an international competition is a delicate one in which fitness training must ensure a maintenance and rebuilding process following an intense season to ensure peak condition for the subsequent intense period of matches (12). During this phase there may also be an additional focus on technical and tactical preparation as opposed to intense physical conditioning (13).This is commonly referred to as the taper, which involves reducing the training loads from a previously intense program to optimise recovery and maximise performance (14, 15). Athletes commonly employ supplementation methods to enhance any adaptations from training routines. Beta-alanine is an amino acid that is ingested over several weeks to increase muscle carnosine content (16) and improve exercise capacity and performance (17). It is considered an effective ergogenic aid by the International Olympic Committee (18), although the effects of beta-alanine on football-specific protocols is unclear and contradictory. Beta-alanine has previously improved YoYo IR2 performance in amateur male footballers throughout a competitive season (19), although YoYo IR1 was not improved in young elite male basketball players (20). Evidence to support beta-alanine supplementation during shorter-duration repeated sprints is distinctly lacking (21–23) although supplementation alongside plyometric training did lead to greater improvements in RAST than training alone in female soccer players (11). This suggests that the combination of training and beta-alanine may be additive, something previously demonstrated with cycling sprint training (24), although no study to date has investigated the combined effect of a football-specific training period alongside beta-alanine supplementation on football-specific performance in females.

The aim of this study was to investigate the effect of beta-alanine supplementation throughout a short-duration intense football-specific training period prior to an international competition on measures of high-intensity running performance. We hypothesised that supplementation would lead to greater improvements in exercise measures than any seen with training alone.

## Methods

### Participants

Twenty-four elite international under-20 (U20) female footballers (age 18 ± 1 y, height 1.67 ± 0.07 m, body mass 62.7 ± 7.4 kg) from different clubs competing in the elite divisions of the Brazilian football pyramid that were part of the national Brazilian team preparing for the South American U20 Women's Championship, volunteered for the study and were randomly assigned to receive either beta-alanine (BA, *N* = 12) or placebo (PL, *N* = 12). Subjects had not taken any creatine supplement in the 3 months prior to the study and had not taken BA for at least 6 months. None of the subjects were vegetarian and, therefore, would have encountered small amounts of beta-alanine in their diet from the hydrolysis of carnosine and its methyl derivatives in meat. The study was approved by the institution's Ethical Advisory Committee.

### Experimental Design

All athletes in this study routinely performed the exercise protocols as part of standard fitness testing throughout their respective seasons. Participants performed the YoYo IR1, RAST, and 20-m sprint test on two separate occasions, separated by 3 weeks of training and supplementation. The exercise tests were performed in a standardized order: Sprint Test and the YoYo IR1 in the morning and the RAST test in the evening. All players performed the same 3-week standardized training program, which consisted of 1 to 2 training sessions per day and received 5 meals per day at standardized timepoints. Sleeping and waking times during the training period were controlled and identical for all athletes. Training and diet, including caffeine consumption, in the 24 h period prior to the first main exercise session were standardized and the athletes repeated this prior to the second main session.

Supplement group allocation was conducted in blocks with groups being equalized according to performance in the YoYo IR1. Throughout the same 3-week period, participants were supplemented with either 6.4 g·day^−1^ of beta-alanine (CarnoSyn^TM^, NAI, USA) or placebo (maltodextrin; NAI, USA) in sustained-release tablets. The dosing regimen consisted of two 800 mg BA or PL tablets ingested four times per day (7 AM/12 PM/5 PM/10 PM). Participants ingested the supplements alongside their standardized meals and a final dose before bed, ensuring all had 100% compliance to the supplementation regimen. No participant in either group reported any symptoms of paraesthesia throughout supplementation.

### Experimental Procedures

#### YoYo Intermittent Recovery Test – Level 1

The YoYo IR1 consists of repeated 2 × 20 m runs between markers at progressively increasing speeds dictated by an audio signal. At the end of each 2 × 20 m bout, individuals performed 10 s of active recovery between consisting of a 10 m (2 × 5 m) walk. The test ended if the player failed to reach the finish line within the given time frame on two consecutive occasions or if the player felt unable to continue (volitional exhaustion). The total distance covered (m) during the test was recorded as the outcome measure.

#### Running Anaerobic Sprint Test (RAST) and 20-m sprint

The RAST consisted of seven 20-m maximal sprints with a passive 10 s recovery period between each sprint; the start of each sprint was indicated by a beep from the photocell equipment (CEFISE standard photocells, Brazil) which measured run time for every individual sprint. The photocells were connected to a computer with specific software (CEFISE, Brasil) for speed analysis. Outcome measures were sprint time of each sprint (s), total sprint time (s), mean, maximum and minimum power output (W) [calculated as Power = (Body Mass × Distance^2^)/Time^3^] and fatigue index (FI, %) [calculated as FI = (peak power - minimum power/peak power) × 100)] (10). Participants also performed 3 separate attempts of a maximum 20 m sprint test, with 5 min passive recovery between efforts. To start the sprint, the volunteer was positioned 1 m behind the first photocell to prevent premature activation of the timer. The timing of the start of each maximal sprint was determined by the athlete.

### Data Analysis

Data were analysed using the SAS statistical package (SAS® University Edition, SAS Institute Inc., USA), and are presented as mean ± 1SD unless stated. Exercise data were analysed using mixed model analysis with individuals assumed as a random factor and supplementation (2 levels; BA and PL) and time (2 levels; Day 0 and 20) assumed as fixed factors. Repeated sprints during the RAST were analysed using mixed model analysis with individuals assumed as a random factor and supplementation (2 levels; BA and PL), time (2 levels; Day 0 and 20) and sprint number (7 levels; 0-7) assumed as fixed factors. Tukey–Kramer adjustments were performed when a significant F value was obtained, and the significance level was set *a priori* at *P* ≤ 0.05. Individual responses for the YoYo IR1 were calculated according to time-to-completion using the spreadsheet of Swinton et al. (25) using 90% confidence intervals, a typical error calculated from reproducibility data (26) and a smallest worthwhile change of 0.2 × the standard deviation of the control session (27). Due to issues unrelated to the intervention (due to minor illness or injury, the coaches instructed the athletes not to complete all protocols as a precautionary measure), complete data for the YoYo IR1 was obtained for 20 athletes (BA = 10, PL = 10) and 22 athletes completed the 20-m sprint (BA = 11, PL = 11); all athletes completed the RAST pre- and post-supplementation.

## Results

### YoYo IR1

**Figure 1:**
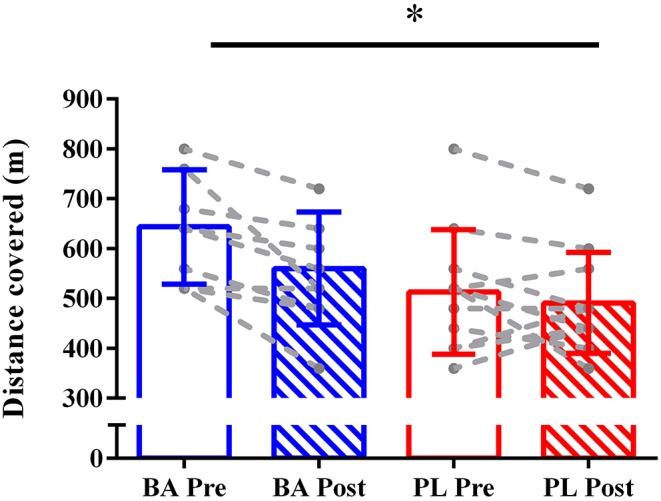
Distance covered during the YoYo IR 1 test in the beta-alanine (BA) and placebo (PL) groups pre- and post-supplementation. *P = 0.004. Effect of time.

YoYo performance was not significantly different between groups at baseline (BA: 644 ± 114 m, PL: 513 ± 125 m; *P* = 0.07), although this almost reached statistical significance. This might be due to missing data (2 individuals from BA and 2 from PL). There was a main effect of group (*P* = 0.046), with lower overall values in the PL vs. BA group, and time (*P* = 0.004); distance covered was lower post- *versus* per-supplementation (−7.4 ± 14.4%). The group × time interaction did not reach statistical significance (*P* = 0.07; Figure 1). Individual data analysis revealed that no individuals in either group improved performance above the smallest worthwhile change during the YoYo IR1, although two athletes in BA and one in PL worsened performance.

### RAST and 20 m Sprint

**Figure 2:**
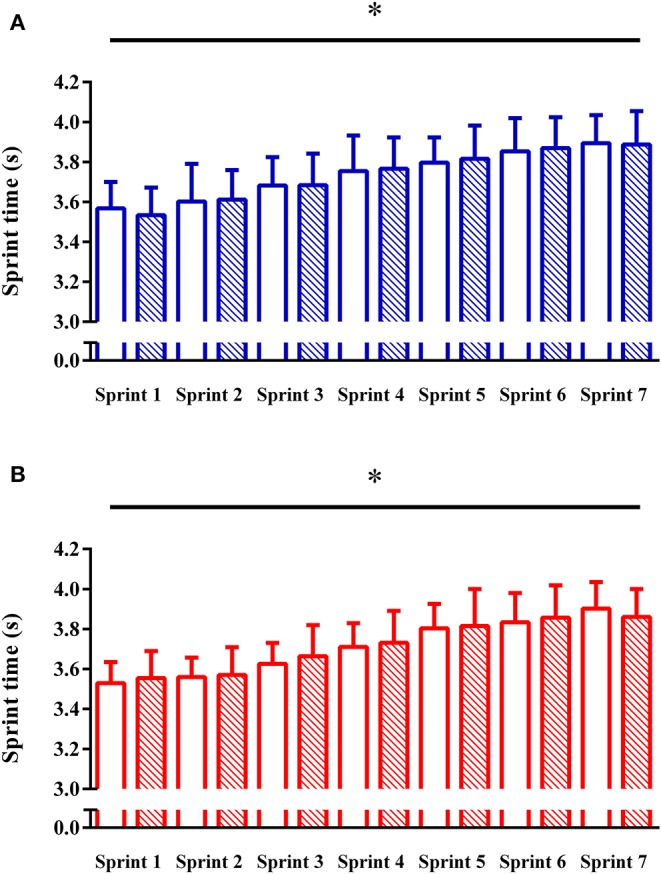
Sprint performance during the RAST for the beta-alanine **(A)** and placebo **(B)** groups pre- (clear bars) and post- (striped bars) supplementation. *P < 0.0001. Effect of sprint number.

There was no effect of group (*P* = 0.67) or time (*P* = 0.45) for sprint times during the RAST, but there was an effect of sprint number (*P* < 0.0001), reflecting an increase in time to complete each sprint with increasing sprint number (Table 1). There were no group × time × sprint interaction effects for sprint times (*P* = 0.96). There were no group, time or group × time interactions for total time, maximum, mean and minimum power and fatigue index during the RAST (all *P* > 0.05) (Figure 2). There was no effect of group (*P* = 0.91), time (*P* = 0.50) or group × time interaction (*P* = 0.25) for the 20-m sprint test.

**Table 1:** Sprint times (s) during the RAST for the beta-alanine (BA) and placebo (PL) groups pre- and post-supplementation.

	**BA**		**PL**	
	**Pre**	**Post**	**Pre**	**Post**
Sprint 1 (s)	3.6 ± 0.1	3.5 ± 0.1	3.5 ± 0.1	3.6 ± 0.1
Sprint 2 (s)	3.6 ± 0.2	3.6 ± 0.1	3.6 ± 0.1	3.6 ± 0.1
Sprint 3 (s)	3.7 ± 0.1*	3.7 ± 0.2*	3.6 ± 0.1*	3.7 ± 0.2*
Sprint 4 (s)	3.8 ± 0.2*	3.8 ± 0.2*	3.7 ± 0.1*	3.7 ± 0.2*
Sprint 5 (s)	3.8 ± 0.1*	3.8 ± 0.2*	3.8 ± 0.1*	3.8 ± 0.2*
Sprint 6 (s)	3.9 ± 0.2*	3.9 ± 0.2*	3.8 ± 0.1*	3.9 ± 0.2*
Sprint 7 (s)	3.9 ± 0.1*	3.9 ± 0.2*	3.9 ± 0.1*	3.9 ± 0.1*
Total sprint time (s)	26.1 ± 1.0	26.2 ± 1.0	26.0 ± 0.6	26.0 ± 1.0
20-m sprint	3.5 ± 0.1	3.5 ± 0.2	3.5 ± 0.1	3.4 ± 0.1

P < 0.0001 Effect of sprint, *indicates a significant difference from Sprint 1.

## Discussion

This study aimed to investigate the effect of BA supplementation in high-level Brazilian female soccer players during a three-week preparatory training period. The main findings showed that responses to BA supplementation were not different from those obtained with placebo and were unable to avoid decreases in performance during the YoYo IR1, which likely occurred due to high workloads imposed in this preparatory period. There were no changes in repeated or maximal sprint performance.

The training load employed with these athletes resulted in a reduced exercise capacity during the YoYo IR1 (−7.4%). Prior to major events, such as an international tournament, it is common to taper, namely reducing the training load from a previously intense program in order to optimise gains and recovery and maximise subsequent performance (14, 15). Our data suggest that, not only was the training intervention too intense in nature to illicit improvements in exercise capacity, it actually worsened performance which is contrary to the aims of the training. Previous data from an under-20 female football team preparing for the World Cup showed a progressive improvement in YoYo IR1 performance leading up to competition (Tunstall H, personal communication in (6)), although the authors suggest this was reflective of a more focused fitness training schedule and the low starting fitness levels of these female players. In the current study, our athletes all plied their trade for top-level national sides, and most were regular starters for their respective teams. It is possible that a long grueling season took its toll on the players, and that performance was a result of accumulated fatigue over the season and would have reduced over this three-week period regardless of the intense training. In fact, YoYo IR1 performance of these athletes prior to the training and supplementation intervention was lower than that shown previously in elite female footballers (7, 9), which provides support for this theory.

Short-term BA supplementation (3 weeks) was unable to attenuate this training-associated decline in YoYo IR1 performance. The lack of an effect shown here is line with previous research showing no changes in YoYo IR1 in young elite male basketball players with BA supplementation (20), although BA did improve YoYo IR2 (19) in amateur male footballers. The YoYo IR2 is initiated at a higher intensity than the YoYo IR1, with a higher contribution from anaerobic glycolytic pathways increasing the contribution of buffering capacity to performance (9), making it more susceptible to improvements with BA. It is also possible that the relatively short loading period in this study did not meet the threshold necessary for a sufficient increase in muscle carnosine to elicit performance improvements. Smith et al. (28) showed similar improvement in cycling capacity following 3 weeks of high-intensity interval training with both BA and PL, but greater improvements from weeks 3–6 were shown with BA. It is currently unknown what the minimal necessary increase in muscle carnosine is to elicit a performance improvement (29) and any definitive conclusions here are not possible due to the lack of muscle carnosine content analysis in the current study. Had the athletes commenced supplementation prior to the training phase, thus ensuring increased muscle carnosine content prior to the intense training period, it is possible that results may have been different. However, we were unable to enforce the supplementation protocol prior to the international period during which we had access to the players, a potential consequence of working with elite club players on international duty. As it stands, short-term BA supplementation was unable to attenuate the decline in YoYo IR1 performance following the training period in this study.

Neither training nor supplementation led to changes in repeated sprint ability or sprint performance. These data are in line with previous studies showing no effect of BA on short-duration repeated sprints in team sports players (21, 22). However, previous research in female football players has shown BA supplementation to improve mean power output during repeated 30-s Wingate sprints (30) and induce greater improvements in repeated-sprint tests when combined with plyometric training compared to training alone (11). It is possible that the highly trained nature of our athletes contributed to these results, since meta-analytical data has shown well-trained individuals to achieve smaller performance gains with supplementation than non-trained individuals (17). The aforementioned studies recruited university level (30) and amateur (11) players, while we employed elite youth players. It is also important to again emphasise that the lack of any changes in these tests may similarly be due to the intense nature of the training program, inhibiting any potential adaptations with or without supplementation.

One of the strengths of this study is that it was a real-world intervention in which we implemented a double-blind placebo-controlled supplementation protocol in top-level female athletes who were part of a competitive international set-up performing their normal pre-competition training program. The standardized pre-tournament training camp provided a unique environment that required all athletes to undergo identical daily routines such as training, nutritional intake and sleep, thus removing several variables which could contribute to individual variability. Indeed, our data showed striking consistency with all but one individual in BA showing a reduction in distance covered during the YoYo IR1, while six athletes in PL also covered less distance; however, statistical analysis revealed that only two in BA and one in PL could be considered to have worsened performance with >90% certainty (25). The controlled nature of this study has great practical applicability to similar athletes undergoing these types of intervention by showing BA to be ineffective during such a short and intense training period. However, alternative methods might be implemented, such as lighter training loads and earlier implementation of the supplementation regime, which might lead to different results. Indeed, this study also highlights the delicate nature of working with an international team since we could not make any changes to their usual routines until their individual seasons with their respective national clubs had ended.

## Practical Applications

Intense preparatory training periods prior to international competitions may place unnecessary strain on top-level footballers following a grueling season. Our data suggest that international teams may inadvertently overload their players leading to a reduced high-intensity exercise capacity in these players. Reduced exercise capacity was not counteracted by short-duration BA supplementation, although it is possible that the supplementation protocol was sub-optimal. Perhaps more communication between clubs and international teams may facilitate this process and avoid overreaching or overtraining, while prior initiation of the supplementation protocol would further benefit any potential adaptation.

## Conclusions

Top-level female footballers involved in this intense 3-week training period prior to a competition worsened their high-intensity intermittent exercise capacity, and this negative result was not attenuated by a short-duration BA supplementation protocol throughout the same period. Further work is necessary to elucidate whether adapted training protocols and BA dosing regimens could lead to better results.

## Data Availability Statement

The datasets generated for this study are available on request to the corresponding author.

## Ethics Statement

The studies involving human participants were reviewed and approved by University of São Paulo. The patients/participants provided their written informed consent to participate in this study.

## Author Contributions

GR, AR, EP, VC, MB, PG, BG, and BS contributed to the conception and design of the study. RR, EP, and BS organized the database and performed the statistical analysis. RR, BD, AG, EP, and BS wrote the first draft of the manuscript. GR, AR, VC, MB, PG, and BG contributed to the subsequent versions. All authors contributed to manuscript revision, and read and approved the final submitted version.

## Conflict of Interest

BS has previously received financial support from Natural Alternatives International (NAI), a company that produces BA, to undertake a study unrelated to this one. NAI has also provided BA supplements free of charge for this and further experimental investigations and supported open access page charges for numerous publications involving the authors. NAI have not had any input (financial, intellectual, or otherwise) into this original investigation. The remaining authors declare that the research was conducted in the absence of any commercial or financial relationships that could be construed as a potential conflict of interest.
